# Spatial Analysis of Chinese American Ethnic Enclaves and Community Health Indicators in New York City

**DOI:** 10.3389/fpubh.2022.815169

**Published:** 2022-01-28

**Authors:** Qiuyi Zhang, Sara S. Metcalf, Harvey D. Palmer, Mary E. Northridge

**Affiliations:** ^1^College of Architecture and Urban Planning, Fujian University of Technology, Fuzhou, China; ^2^Department of Geography, University at Buffalo, Buffalo, NY, United States; ^3^Department of Political Science, University at Buffalo, Buffalo, NY, United States; ^4^Hansjorg Wyss Department of Plastic Surgery, NYU Grossman School of Medicine, New York, NY, United States

**Keywords:** ethnic enclaves, Chinese Americans, New York City, community health, residential segregation

## Abstract

In New York City, the population of Chinese Americans has grown faster than that of any other minority racial/ethnic group, and now this community constitutes almost half of all Chinese Americans living in the northeastern United States. Nonetheless, scant research attention has been given to Chinese American ethnic enclaves and little is known about the health status of their residents. This study aims to help address this gap in the literature by: (1) improving our understanding of the spatial settlement of Chinese Americans living in New York City from 2000 to 2016; and (2) assessing associations between a New York City resident's likelihood of living in a Chinese American enclave and their access to health care and perceived health status, two measures of community health. In support of this aim, this study establishes a robust criterion for defining ethnic enclaves at the Census tract level in New York City as the communities of interest in this paper. An ethnic enclave is defined as an area at the Census tract level with high dissimilarity and a spatial cluster of Chinese Americans. The spatial findings were that Chinese Americans in New York City were least segregated from other Asian American residents, somewhat segregated from White residents, and most segregated from Black residents. Also, the population density of Chinese Americans increased since 2000, as reflected by their declining exposure index with other Asian Americans. Results from logistic regression indicated that the probability of living in a Chinese American enclave was negatively associated with positive self-perception of general health and positively associated with delays in receiving health care. For Chinese American residents of New York City, living in an ethnic enclave was also associated with both lower socioeconomic status and poorer community health.

## Introduction

Since 2000, the United States Census reported Asian American population grew faster than any other racial minority group and the Chinese population is the largest ethnic Asian subgroup, comprising 25.9% of the Asian American population as of 2010. This study examines whether patterns of residential settlement are associated with health-related factors for Chinese American communities in New York City. New York City is home to the largest Chinese American population outside of Asia (1), representing almost half (47%) of Chinese Americans living in the Northeastern United States (2). This population is not only large but rapidly growing relative to other racial/ethnic minority groups. According to the U.S. Census Bureau (3–8), Chinese Americans living in New York City increased by 63 percent from 2000 to 2016 (from 361,531 to 590,340 people).

Previous research on immigration and assimilation has suggested that the ethnic enclaves that often emerge from immigrant settlement are sustained over time when they function to support access to affordable housing, strengthen family ties and cultural identity, and help with finding job opportunities (9, 10). The term “ethnic enclave” refers to a geographical area where a particular minority ethnic group is spatially clustered in such a way that the group is socially and economically distinct from the majority group (11). These enclaves refer not only to physical settings, but also to established neighborhoods in locations desirable to the minority group where multiple generations reside. One early study, for example, found that residential self-segregation was typical of “middleman” minorities that effectively resist assimilation by forming highly organized communities (12). This self-segregation process is reflected in the concept of “ethnic community,” a term that has been applied to characterize the satellite Chinatowns that have arisen in Flushing and other outlying parts of the New York City metropolitan region (13).

A large body of social science and public health research has documented links between racial/ethnic residential segregation and health-related factors among racial/ethnic minority populations in the US (14–18). In addition, previous research has surveyed the health concerns and needs of Asian Americans in New York City, such as the community's perceived health status, health-seeking behaviors, barriers to care, and level of available health resources (19). Analysis of the CHNRA data found that a large plurality (48%) of Chinese American respondents described their health status as “fair or poor” compared to 30% of New York City Asian Americans overall and with 23% of all New York City residents (19). Similarly, many Asian Americans experience barriers to healthcare such as language differences, financial limitations, cultural factors that influence access and choice, and more limited transportation options (19–21). Little is known, however, about potential associations between ethnic enclaves and the health of Chinese Americans. This gap in knowledge arises from the unclear boundaries for ethnic enclaves and the limited availability of individual health data for the many different Asian American minority groups.

Previous research has examined Asian American enclaves in New York City based on analysis at the community district level and found that enclave residence was associated with positive self-perceptions of health (11). However, associations between enclave residence and health-related behaviors, such as smoking, were harder to identify. Due to the cultural and economic diversity that exists within the Asian American population cohort, especially evident in differences between East Asians and South Asians, a binary distinction for Asian ethnicity is insufficient to properly capture such a wide range of potential associations with health behavior factors (11). Another concern about this research design is that the community district may be too large of a spatial unit, where the level of aggregation is too high to sufficiently distinguish among Asian American enclaves for the purpose of identifying differences in health status and care. This concern is generally expressed as the modifiable areal unit problem (MAUP), referring to limitations that arise from failing to take into account the effect of spatial scale and the zoning scheme in determining the unit of analysis (22–25). The MAUP concern indicates that as the size and shape of areal units change, so do the relationships that can be observed, such as the size and direction of observed regression and correlation coefficients (23, 26).

When considering the contextual determinants of health behaviors and outcomes, one solution to the MAUP is to minimize the modifiability of the geographic unit of analysis (27–29). Using spatially aggregated Census data to evaluate segregation in residential areas of a region or a city has been a standard approach (30). Some studies have shown how segregation level varies by neighborhoods using local measures (14, 31, 32). Other studies have demonstrated how racial/ethnic residential segregation may have impacts on health outcomes in New York City (33–35).

The present study addresses these methodological issues by improving the specification of ethnic enclaves and then estimates the association between the probability of living in a Chinese American enclave and factors related to the health of community residents, that is, community health. Two research questions in particular motivated this study:

1) How did patterns of residential settlement change for Chinese Americans living in New York City from 1980 to 2016? We hypothesized that spatio-temporal patterns of Chinese American settlement in New York City were concentrated in ethnic enclaves. Answering this question required establishment of a criterion for defining Chinese American ethnic enclaves at the Census tract level.

2) What kind of associations exist between the probability of living in a Chinese American enclave and community health? The answer to this question depends in part upon which health factors are considered. We expect that residents of these Chinese American enclaves experience barriers to achieving positive health outcomes, resulting in negative associations overall, i.e., poorer community health. However, we recognize that enclaves also have the potential to provide protective health benefits through social support, so the associations for some health factors could be weak or even positive, resulting in improved community health.

Geospatial information was combined with population attribute data using Geographic Information Systems (GIS) to answer these research questions, as detailed in the next section.

## Data and Methods

Spatial statistical analysis was performed to examine residential settlement patterns of Chinese Americans living in New York City from 1980 to 2016. Data from the U.S. Census Bureau Decennial Census (DEC 1980, 1990, 2000, 2010) and the American Community Survey (ACS 2010, 2016) were used to conduct this analysis (3, 4, 8). To assess associations between ethnic enclaves and community health indicators, data were obtained from local health surveys such as the New York City Department of Health and Mental Hygiene Community Health Survey (CHS 2011-2013) (36). See Appendix for a full listing of the variables used in this study and their sources.

**Figure 1:**
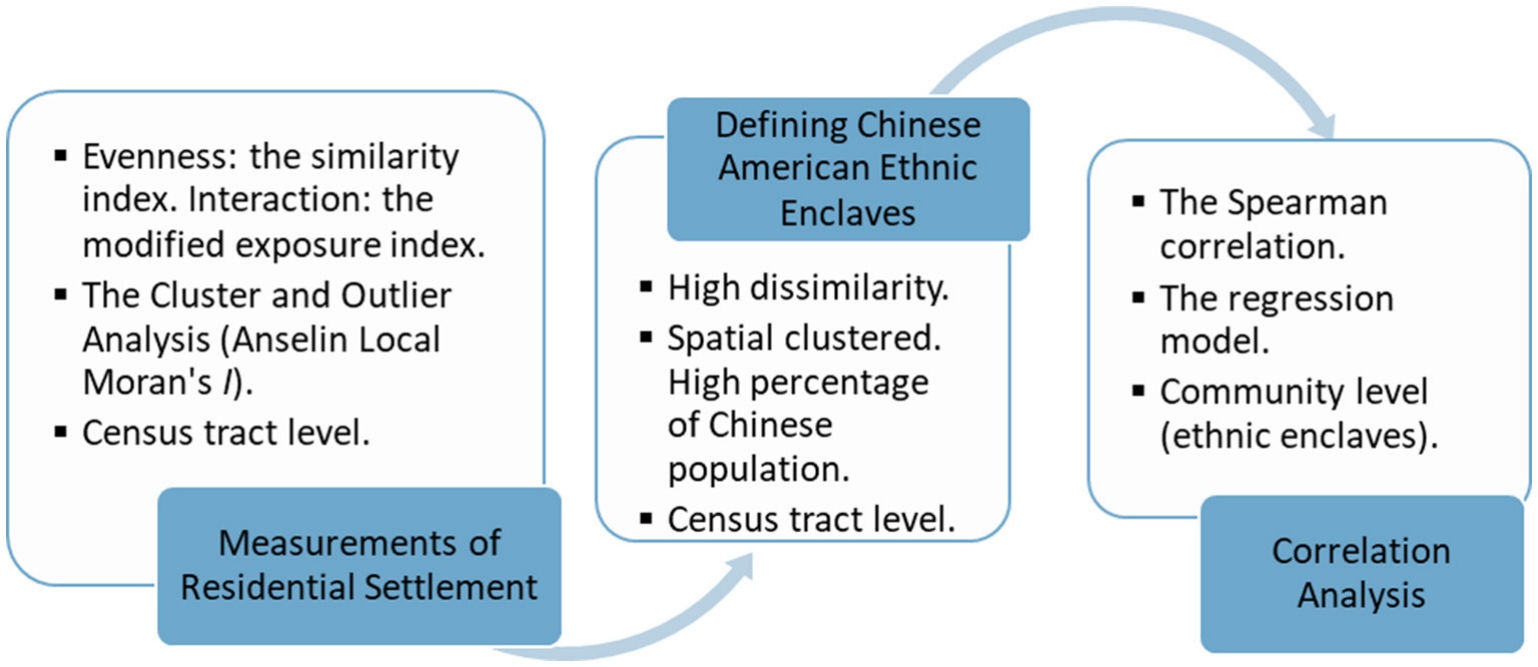
The methods and process flow for this study.

Figure 1 provides an overview of the methods and process flow for this study, in 3 main steps: measuring patterns of residential settlement, defining Chinese American ethnic enclaves, and using this information to conduct correlation analyses of statistical associations. These steps are described in the sections that follow.

### Data Collection

In order to understand patterns of Chinese American residential settlement and define the boundaries of Chinese American enclaves in New York City, the spatially explicit data derive mainly from the U.S. Census Bureau American Community Survey (ACS) and Decennial (DEC) from 1980 to 2016 (3–8). Due to data limitations precluding analysis of Chinese American data in earlier years, this study uses Asian American population data instead of Chinese American population data for the years prior to 2000. For analyzing associations between Chinese American enclaves and community health indicators, the data derive mainly from the U.S. Census Bureau 2012-2016 ACS 5-Year Estimates and the 2011-13 NYC Department of Health and Mental Hygiene (DOHMH) Community Health Survey (CHS) (4, 36). All variables used in this study were standardized to a proportion from 0 to 1, where 1 corresponds to 100% of the population. A full description of the variables used in this study is provided in the Appendix.

The community health indicators used in this study were obtained from the 2011-2013 NYC DOHMH data, as follows: Perception of Health, Smoking, Sugary Drink, and Delay in Receiving Healthcare (36). Values for other variables were obtained from 2016 ACS data (4), including population, the proportion of Chinese Americans and Whites in the population, age-related variables, and the proportion of the population in poverty, with full time employment, and with limited English. Additional independent variables were generated from modifications to the ACS data as detailed in the Appendix.

**Figure 2:**
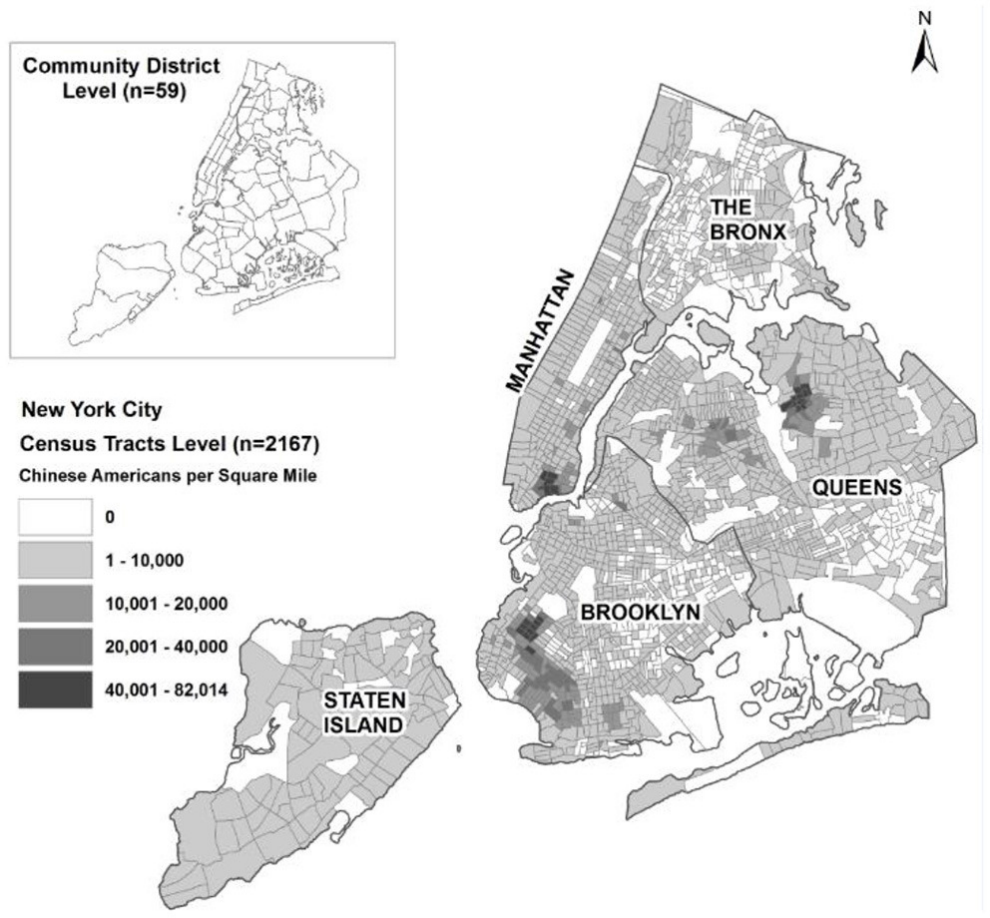
Distribution of Chinese Americans in New York City in 2016. Source: U.S. Census Bureau (6).

The study area of New York City includes 2167 Census Tracts and 59 residential Community Districts (see Figure 2). The map in Figure 2 illustrates Chinese American population density by Census tract as distributed across the five boroughs (Manhattan, The Bronx, Queens, Brooklyn and State Island) of New York City. Census tracts are the finest resolution of spatial unit analyzed in this study.

### Measurements of Residential Settlement

Statistical indicators, including the similarity index and the modified exposure index, were applied in this study to U.S. Census tract-level data to compare different population groups living in New York City over time. A time series analysis was conducted using U.S. Census estimates from 1980, 1990, 2000, and 2010 (8). The similarity index (*S*) ranges from 0 (complete segregation) to 1 (no segregation) and is used to reflect the level of segregation of Chinese Americans from mainstream U.S. society. A higher value for the similarity index may be interpreted as meaning that there are more potential interactions with members of other ethnic groups (37, 38). The similarity index, *S*, can be defined in terms of the dissimilarity index, *D*, as follows: *S* = 1 – *D*.

Studies of residential settlement often use the dissimilarity index (39) to measure the level of segregation among population groups and the exposure/isolation index (40) to measure the level of interaction among population groups. For assessing segregation between Chinese Americans and White Americans in New York City, the dissimilarity index *D* is given by equation 1 below:


(1) $$\begin{array}{r} D=1/2\underset{i=1}{\sum }|\;\frac{w_{i}}{W}-\;\frac{c_{i}}{C}\;| \end{array}$$

where w_i_ = population of White Americans in Census tract *i*

c_i_ = population of Chinese Americans in Census tract *i*

W = total population of White Americans in New York City

C = total population of Chinese Americans in New York City

The exposure index *E*_*CW*_ measures the degree of exposure of Chinese Americans to White Americans at the US Census tract level, relative to the New York City-wide level of exposure of Chinese Americans to White Americans. *E*_*CW*_ refers to segregation from a sociological perspective, i.e., the probability of contact of one ethnic group with another ethnic group within a spatial context (38, 41). When there are only two ethnic groups, its theoretical range is from 0 to 1, where 0 indicates complete balance or no segregation, e.g., Chinese Americans would encounter White Americans in their Census tract at a rate equal to that at the New York City-wide level. At the other extreme, 1 indicates complete isolation or segregation, e.g., Chinese Americans would come into contact with only other Chinese Americans (37).

Exposure of Chinese Americans to White Americans, *E*_*CW*_, is given by the expression in Equation 2 and is calculated in the same manner for exposure of Chinese Americans to other ethnic groups:


(2) $$\begin{array}{c} E_{CW}=1-\;\frac{\sum_{i}c_{i}w^{\prime }{}_{i}}{CW^{\prime }} \end{array}$$

where w_i_' = proportion of the White American population in Census tract i;

c_i_ = number of Chinese Americans in Census tract i;

W'= proportion of the White American population in New York City;

C = number of Chinese Americans in New York City.

In a multiethnic setting such as New York City, however, the theoretical minimum of *E* for any pair of ethnic groups is 1 – 1/(p_1_ + p_2_) where p_1_ and p_2_ are the proportions of the city population in ethnic groups 1 and 2, respectively. This minimum will be less than 0, since (p_1_ + p_2_) <1 when there are more than 2 ethnic groups, and becomes more negative as p_1_ + p_2_ gets smaller, e.g., in 2016 New York City, it is −1.011 when E is calculated for White and Chinese Americans compared to −6.296 when calculated for Chinese and other Asian Americans. For this reason, a modified exposure index (E') is used to ensure that all values are positive (37). This index is related to the exposure term, *E*', as per the expression in Equation 3:


(3) $$\begin{array}{r} {\text{E}}^{\prime }=1-\text{E} \end{array}$$

Given this relationship, E' = 0 indicates complete segregation of the two ethnic groups from each other, E' = 1 depicts when the Census tract exposure level is equal to the New York City-wide level of exposure, and *E'* > 1 can occur if the Census tract exposure level is greater such that the two ethnic groups are segregated together away from other ethnic groups in New York City. As with the similarity index, the modified exposure index may be interpreted as meaning that higher values of the index indicate more potential interactions with members of another ethnic group.

### Spatial Clusters and Outlier Analysis

The Census tract-based residential settlement measures described above are regarded as spatial either because they explicitly utilize geographical information in their formulations, such that the results will change if the locations of population groups have changed, or because the spatial interaction among population groups across areal unit boundaries is accounted for in determining the level of segregation (42). An advantage of using a geographic information system (GIS) is the spatial analytical capability that it provides. GIS supports analysis of spatial features by combining geographical information and attribute data. In this study, the Global Moran's *I* and Anselin (43) Local Moran's *I* were used to detect spatial clusters and outliers for the population density of Chinese Americans. To do this, the ArcGIS software tool was used to calculate global and local Moran's *I* values, z-scores, pseudo *p*-values for those index variables, and summary coding (“High-High” or “Low-Low”) denoting the cluster type for each statistically significant feature (44).

Through the spatial analysis, algorithms were designed to construct the residential settlement measures. These algorithms consisted of the general procedures to extract spatial information from Census data and to combine this spatial information with attribute (population) data in order to derive the indices. Chinese American enclaves were defined as areas with high dissimilarity, high population density, and spatial clusters of Chinese American residents.

### Correlation Analysis of Statistical Associations

#### Spearman Correlation

Analysis of the patterns of residential settlement was used to reveal which Census tracts have higher concentrations of Chinese Americans. Given that New York City is a multiethnic setting, these Chinese American enclaves were compared to Census tracts with large White majority populations. The Spearman rank correlation method measures the strength of the linear relationship between variables (45). It is an apt non-parametric test to determine whether there are correlations between the percent of Chinese Americans in a community and the residents' health and healthcare-related factors (such as insurance coverage, self-reported health conditions, consumption of sugary beverages, smoking behaviors, and need for medical care), and a series of demographic and socioeconomic factors (such as highest education level attained, poverty status, limited English proficiency, and foreign-born status). All the variables were bounded between 0 and 1, representing the proportion of the population in each category at the Census tract level.

#### Regression Model

As a means of focusing the analysis on what distinguishes Chinese American enclaves while allowing for multivariate correlation, regression models were developed to compare the characteristics of Census tracts with higher Chinese American concentrations to those with large White majority populations. The dependent variable was coded “1” if the Census tract is a Chinese American ethnic enclave and “0” if it is a non-ethnic enclave. The independent variables were the socio-demographic and health-related characteristics of the Census tract's population. The traditional method for a binary dependent variable is the binomial logistic model, which often fits the data better than the linear model, and the logistic model assumes that the natural log of the odds ratio is a linear function of the regressors. So, the binomial logistic model was applied in this study.

## Results

### Residential Settlement of Chinese Americans

**Figure 3:**
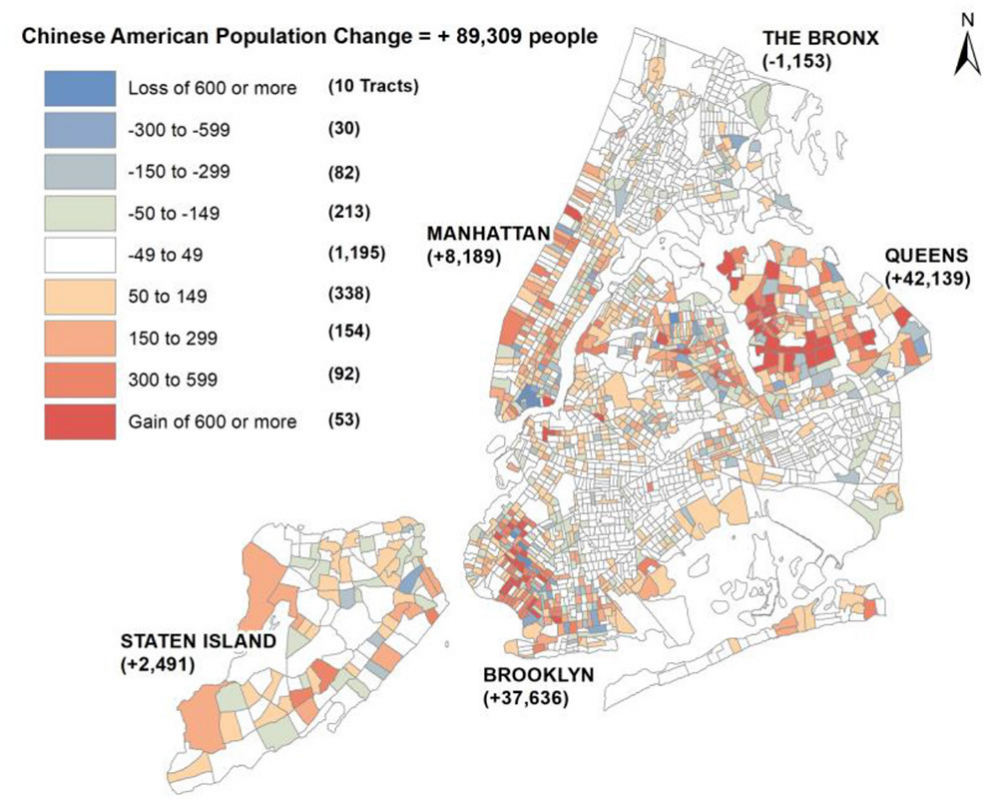
Change in the Chinese American population from 2010 to 2016. Source: U.S. Census Bureau ACS 2010, 2016 (3, 4, 6).

Based on analysis of the US Census data presented in Figure 3, the population of Chinese Americans in New York City increased by 19% (89,309 people) from 2010 to 2016. The two boroughs that experienced the greatest increases in Chinese American residents were Queens and Brooklyn, which increased 22% (42,139 people) and 23% (37,636 people), respectively. This analysis indicated growth of Chinese American enclaves in Brooklyn and Queens rather than in the historic Manhattan Chinatown neighborhoods, which experienced a decline in Chinese American residents over this time period.

A study hypothesis is that there is a high level of residential segregation among Chinese Americans living in New York City. To test this hypothesis, the similarity index and the modified exposure index were applied to Census tract level data for 1980, 1990, 2000, 2010 and 2016 (6, 8). Analysis of these metrics revealed that Chinese American segregation was most severe relative to the population of Black / African Americans, followed by that of Whites, and least severe in comparison with other Asian Americans (see Table 1). From 1980 (DEC) to 1990 (DEC), Asian Americans as a group were most segregated from Black / African Americans and least segregated from Whites. From 2000 (DEC) to 2010 (DEC), the similarity indices declined for Whites and other Asian Americans but remained stable with Black / African Americans, indicating increases in residential segregation of Chinese Americans. From 2010 (ACS) to 2016 (ACS), the similarity indices increased for Whites and Blacks but remained stable for other Asian Americans, indicating decreases in residential segregation of Chinese Americans relative to these populations.

**Table 1:** Similarity and modified exposure indices for Chinese Americans and Other Racial/Ethnic Groups in New York City, 1980 - 2016.

	**1980-DEC (Asian)**	**1990-DEC (Asian)**	**2000-DEC (Chinese)**	**2010-DEC (Chinese)**	**2010 ACS (Chinese)**	**2016 ACS (Chinese)**
Similarity index (**S**)						
White	0.53	0.55	0.44	0.42	0.41	0.43
Black	0.21	0.22	0.14	0.14	0.13	0.15
Other Asian			0.51	0.49	0.46	0.46
Modified exposure index (**E'**)						
White	1.03	1.07	1.05	1.04	1.00	0.97
Black	0.44	0.39	0.23	0.24	0.24	0.26
Other Asian			1.68	1.54	1.53	1.44

The modified exposure indices for Chinese Americans relative to Whites are ~1, indicating that the exposure of Chinese Americans to Whites at the Census tract level is equal to the citywide level of exposure of Chinese Americans to Whites. Lower values of the modified exposure indices may be interpreted as lower levels of exposure of Chinese Americans to a given racial/ethnic group at the Census tract level, as compared to the citywide level of exposure. The results (see Table 1) indicate that Chinese Americans in New York City have the most exposure to other Asian Americans, moderate exposure to Whites, and least exposure to Blacks.

### Pattern Analysis of Ethnic Enclaves

In order to test whether Chinese American residents were randomly distributed among Census tracts in New York City, the global Moran's *I* was applied to assess the overall clustering pattern and trend in the data from 2000 to 2016 (see Table 2). Findings were that values for the index increased significantly (*p* = 0.01) over the time period, indicating an increased spatial autocorrelation for Chinese Americans.

**Table 2:** The overall pattern of Chinese American population density in New York City, 2000 - 2016.

**Dataset**	**Global Moran's I**	**No. of High-High tracts**	**Area (square miles)**	**Average Chinese Americans per square miles**
2000 DEC	0.31	312	24.23	10,804
2010 DEC	0.39	328	25.80	13,780
2010 ACS 5 YR	0.37	309	24.34	13,838
2016 ACS 5 YR	0.44	317	25.76	15,542

**Figure 4:**
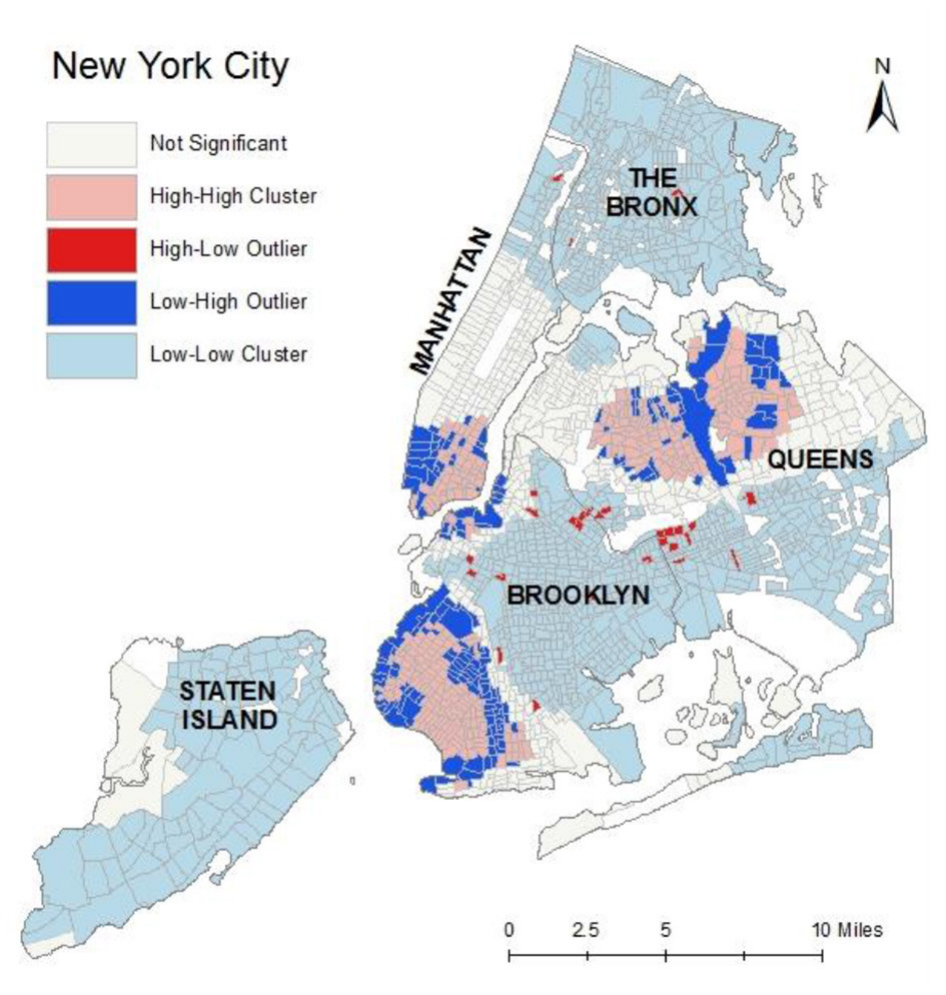
The spatial distribution of Chinese American outliers and clusters, 2016.

The Anselin (39) Local Moran's *I* was applied to assess the specific spatial distributions of Chinese American residents (see Figure 4). High-High and Low-Low tracts indicate statistically significant cluster of high and low population density, respectively. If a feature has a high value and is surrounded by features with low values, it is termed a High-Low outlier. If a feature has a low value and is surrounded by features with high values, it is termed a Low-High outlier. The pink shaded areas in Figure 4 indicate Census tracts with high Chinese American population density surrounded by other areas with high Chinese American population density. These are potential locations of Chinese American enclaves, but our definition also takes into account the level of dissimilarity.

**Figure 5:**
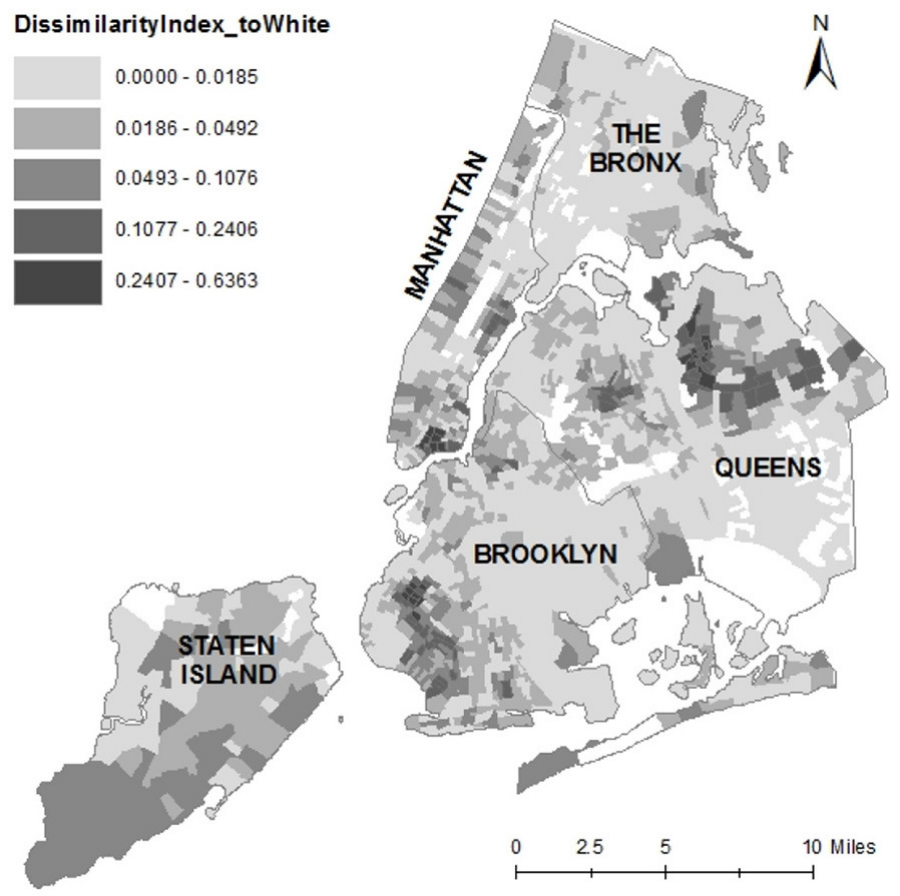
The dissimilarity index map of Chinese Americans to Whites at the Census tract level, New York City.

The value of the dissimilarity index is statistically independent from the relative sizes of the groups used in its computation. Figure 5 illustrates the dissimilarity indices for Chinese Americans relative to Whites at the Census tract level in New York City.

High-High cluster areas (n=317) with relatively high dissimilarity and high percentages of Chinese Americans were chosen to define **ethnic enclave areas** (*Dissimilarity to White* > *0.0133 AND Dissimilarity to Black* > *0.0246 AND Dissimilarity to Other Asian* > *0.0094 AND COType* = '*HH' AND % Chinese Americans* > *36*). This definition of ethnic enclave represents a two-dimensional approach that combines social (dissimilarity index) and physical (spatial clustering, population density) components. The threshold of each dissimilarity index was set to the median value, and the threshold for the percent of Chinese Americans was set to the 95^th^ quantile value (see the bold values in Table 3).

**Table 3:** Descriptive statistics used in defining ethnic enclaves, 2016.

**Description**	**Min**	**Max**	**Mean**	**25^**th**^ Quantile**	**Median**	**75^**th**^ Quantile**	**95^**th**^ Quantile**
% Chinese Americans	0.00	80.00	6.79	0.30	1.90	6.70	**36.0**
The dissimilarity index (D)							
White	0.0000	0.6363	0.0276	0.0043	**0.0133**	0.0318	0.0972
Black	0.0000	0.6476	0.0398	0.0072	**0.0246**	0.0540	0.1241
Other Asian	0.0000	0.6240	0.0307	0.0019	**0.0094**	0.0326	0.1413

*Data Source: U.S. Census Bureau 2016 ACS. Bold indicates the results are significant at p < 0.01*.

**Figure 6:**
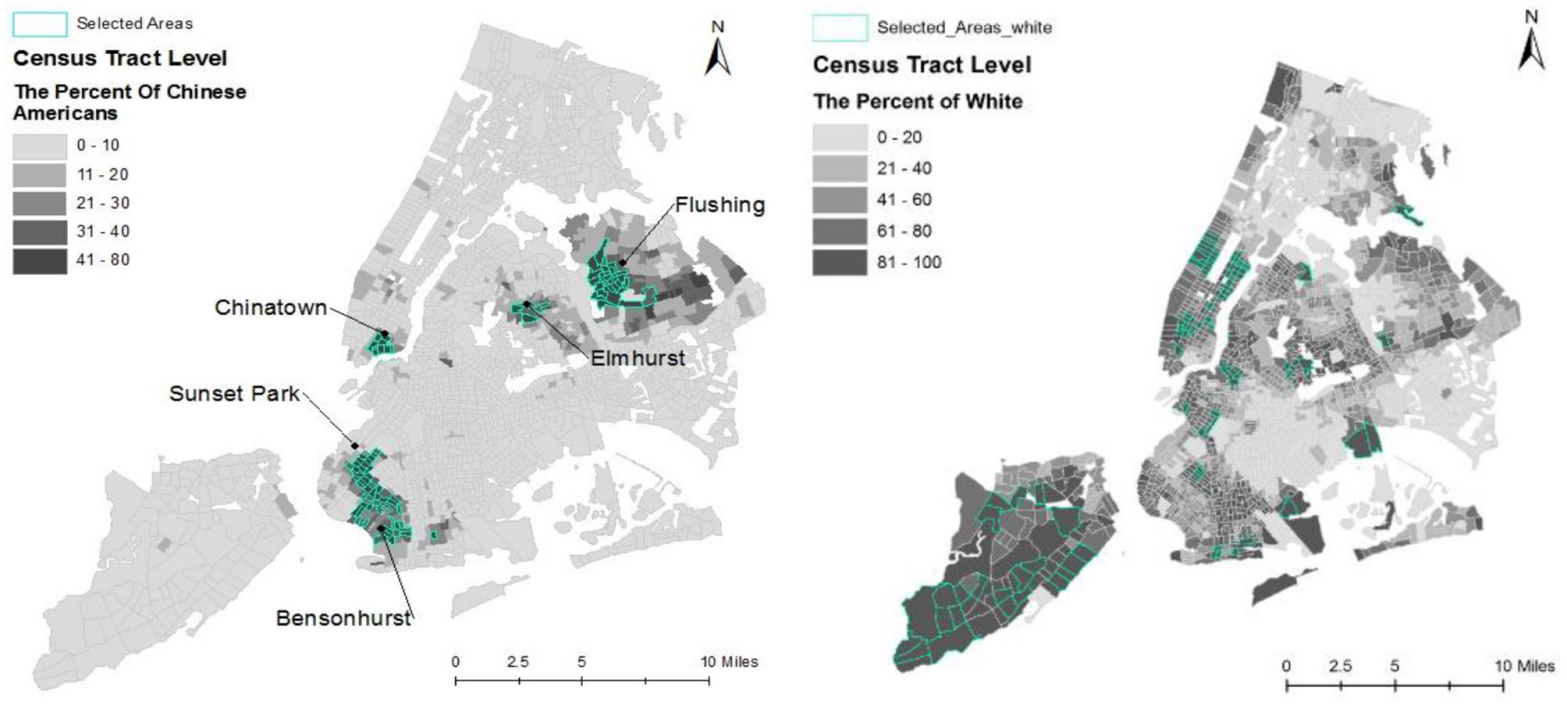
Maps of Chinese American ethnic enclaves (left panel) and non-ethnic enclave areas (right panel), New York City.

After filtering the dataset using these rules, 90 of 2167 Census tracts representing 5 of 59 community districts were defined as ethnic enclaves of Chinese Americans living in New York City (see Figure 6, left panel).

The map in the left panel of Figure 6 indicates that both Sunset Park East in Brooklyn and Flushing in Queens have greater proportions of Chinese American residents than the Chinatown neighborhood in Manhattan. Neighborhoods nearby in Brooklyn and Queens have also grown rapidly, possibly due to an influx of new immigrants and Chinese Americans relocating from Manhattan to these outer boroughs due to lower housing costs.

For comparision (see Figure 6, right panel), and in order to avoid the influence of other minority population groups, this study selected 91 of 2167 Census tracts as **non-ethnic enclave areas** with large proportions of the White majority population (*% White* > *80; Total population* > *5000*). In total, 181 Census tracts were included in the following analysis: 90 Chinese American ethnic enclaves and 91 non-ethnic enclaves. The community health survey data at the community district level were assigned to each Census tract. In total, 63% of Census tracts were located within a single community distrct, 30% of Census tracts were located within two community distrcts, and 7% of Census tracts were located within three or more community distrcts. For the latter two conditions, the average value across the covered community districts was assigned to each Census tract.

### Correlation Analysis

Table 4 presents Spearman correlation coefficients for sociodemographic characteristics correlated with the percentage of Chinese American adults in the 181 selected Census tracts. Comparing Chinese American ethnic enclaves with non-ethnic enclaves, the percentage of foreign-born (0.796) and the percentage of limited English speakers (0.795) indicated a strong positive correlation with the percentage of Chinese Americans. It is indeed reasonable to expect that Chinese Americans are mostly foreign born with limited English proficiency. Findings also indicate that the percentage of Chinese Americans was associated with low socioeconomic status measures, including a positive correlation with poverty (0.551) and a negative correlation with working full-time (−0.368), both of which were statistically significant (*p* < 0.01).

**Table 4:** Spearman correlation coefficients for sociodemographic characteristics and community health indicators correlated with the proportion of Chinese Americans in ethnic-enclave Census tracts.

	**% Chinese Americans**
% With limited English	0.795^**^
% Foreign born	0.796^**^
% With full time employment	−0.368^**^
% In poverty	0.551^**^
% 25–64 years with < HS education	0.731^**^
% 25–64 years with < HS education & private ins.	−0.356^**^
% 25–64 years with < HS education & public ins.	0.110
% 25–64 years with < HS education & no ins.	0.490^**^
% 65+ years with < HS education	0.686^**^
% 65+ years with < HS education & private ins.	−0.278^**^
% 65+ years with < HS education & public ins.	−0.294^**^
% 65+ years with < HS education & no ins.	0.338^**^
% With good perception of health	−0.346^**^
% Who smoke tobacco	−0.017
% Who consume sugary drinks	0.108
% With delays in receiving health care	0.184^*^

* *indicates the results are significant at p < 0.05*;

** *indicate the results are significant at p < 0.01*.

For both age groups (25–64 years old; 65 years and older), the percentage of the population with low educational attainment (less than high school) exhibited a strong positive correlation with the percentage of Chinese American adults. Further, the percentage of the low educational attainment population with private insurance coverage demonstrated a *negative* correlation, for both age groups, with the percentage of Chinese Americans. Similarly, the percentage of the low educational attainment population with *no* insurance coverage exhibited a *positive* correlation with the percentage of Chinese Americans.

As the Chinese American population with low educational attainment ages, such that the community has a smaller percentage 25–64 years old (group 1) relative to the percentage 65 years and older (group 2), the more relevant correlation with the percentage of residents with public insurance shifts from a very weak positive (0.110) to a weak negative (−0.294). This pattern suggests a worsening health-coverage situation for Chinese Americans with low educational attainment as they age, where their likelihood of having public insurance is no different than that for the residents of non-ethnic enclaves when they are middle aged but is lower when they are oldest.

The percentage of the population with a positive self-perception of general health demonstrated a negative correlation (−0.346) with the percentage of Chinese Americans. The percentage of residents who consume sugary drinks and the percentage of residents who smoke, however, exhibited no statistically significant correlations with the percentage of Chinese Americans. Finally, the percentage of the population experiencing delays in receiving medical care demonstrated a weak positive correlation (0.184) with the percentage of Chinese Americans.

Table 5 reports the results for a logistic regression model of Chinese American ethnic enclaves. Given that the independent variables are bounded from 0–1, a one-unit change is the entire range of the variable, so the odds-ratio interpretation of the logit coefficients, *Exp(B)*, is not particularly intuitive, especially given that the analyzed Census tracts are a selected subset so the odds in the ratio are not population rates. A more intuitive but less direct approach is to use the coefficients to calculate probability changes relative to a representative baseline probability of *Y* = *1*. The logit coefficients can be used to calculate the change in the predicted probability of *Y* = *1* from a change in an independent variable holding all of the other regressors constant, which is equivalent to setting the Pr(*Y* = *1*) prior to the change in the independent variable equal to a representative baseline probability. Given that 90 of the 181 Census tracts included in the analysis are Chinese American enclaves, Pr(*Y* = *1*) = 0.50 was used as the baseline (prior) probability for calculating predicted probability changes.

**Table 5:** Results of binary logistic regression model (Pseudo R-square = 0.414, *n* = 181).

	**B**	**S.E**.	**Wald**	**Sig**.	**Exp(B)**	**95% C.I.for EXP(B)**	
						**Lower**	**Upper**
(Constant)	7.720	2.326	11.018	0.001			
% 65+ years	−5.448	2.826	3.717	0.054	0.004	0.000	1.094
% 25–64 years with < HS education & public ins.	0.935	0.773	1.462	0.227	2.546	0.560	11.587
% 65+ years with < HS education & public ins.	−1.320	1.200	1.211	0.271	0.267	0.025	2.805
% With good perception of health	−4.499	1.693	7.060	**0.008**	0.011	0.000	0.307
% Who consume sugary drinks	0.126	4.188	0.001	0.976	1.134	0.000	4159.813
% With delays in receiving health care	31.929	10.591	9.089	**0.003**	7.356x10^13^	71056.901	7.615x10^22^
% With full time employment	−8.773	2.434	12.988	**0.000**	0.000	0.000	0.018

*Bold indicates the results are significant at p < 0.01*.

Overall, the results identify the factors that are significantly correlated with the Census tract being a Chinese American enclave. Relative to non-ethnic enclaves, Chinese American enclaves have populations with a lower percent who hold positive (good or better) perceptions of health, a higher percent experiencing delays in receiving health care, and a lower percent with full-time employment. All of these regressors in the logistic model have coefficients that are highly significant statistically (*p* < 0.01).

When assessing the relative substantive significance of the regressors (with coefficients with *p* < 0.10), differences in their sample distributions should be taken into account by comparing the estimated effects of one-standard-deviation (one-SD) changes rather than fixed-unit changes. Taking that approach, full time employment has the largest substantive impact, accounting for more of the differences between Chinese American enclaves and non-ethnic enclaves, and specifically reducing the predicted probability of a Census tract being a Chinese American enclave by 0.229, from a one-SD (0.113 unit) increase. In comparison, one-SD increases in the proportions of the Census tract population who experienced delays in receiving health care (+0.022 units), have a positive self-perception of health (+0.132), and are over 65 years old (+0.072), produced changes in the predicted probability of a Chinese American enclave of +0.169, −0.144, and −0.097, respectively. Note though that these probability changes are relative to the baseline Pr(*Y* = *1*) = 0.50, so they are (by definition) the maximum effects on Pr(*Y* = *1*) even if they are representative in the current application due to the near even split of Census tracts between Chinese American enclaves and non-ethnic enclaves.

## Discussion

This research provides a novel way to define ethnic enclaves, unique communities that maintain a strong sense of ethnic or national identity within another country, that are hypothesized to possess health profiles that differ from those of more assimilated communities and may be transferrable to diverse ethnic groups. In this study, the similarity of residents living in Chinese American ethnic enclaves and non-ethnic enclaves in New York City were assessed, and the degree of exposure of Chinese Americans to Whites, Blacks, and other Asian Americans were measured. Results indicate that Chinese Americans living in New York City are most segregated from Blacks, somewhat segregated from Whites, and least segregated from other Asian Americans (to the extent that they are segregated with them and away from other minority ethnic groups). Compared to estimates of the similarity and modified exposure indices for Los Angeles County in 2000 (37), Chinese Americans in New York City were less segregated from Whites and less geographically clustered with other Asian Americans but more segregated from Blacks.

The analyses performed in this study using statistical indicators calculated with Census tract data confirmed that the Chinese American population density increased in New York City from 2000 to 2016, as signified by the declining exposure index of Chinese Americans relative to other Asian Americans. These findings may reflect a preference for proximity to other Chinese Americans (given that the exposure index with Whites also declines), ease of transportation access to the ethnic enclaves, and reliance on family and kinship networks to obtain housing and other resources.

From a geographic perspective, this study identified both spatial clusters and outlier areas with concentrations of Chinese American residents. An ethnic enclave was defined in this study as an area with a high percentage of Chinese American population, a high dissimilarity index, and high spatial clustering of Chinese Americans relative to other groups living in New York City. Using this definition, 90 of 2047 census tracts (5 of 59 community districts) were identified as Chinese American enclaves.

For Chinese American adults, living in ethnic enclaves was associated with more negative perceptions of general health, greater likelihood of experiencing delays in receiving health care, lower socioeconomic status, less private insurance coverage for those with low educational attainment (less than high school), and less public insurance coverage among older Chinese Americans with low educational attainment. Similarly, a logistic regression model found that relative to non-ethnic enclaves, Chinese American enclaves have populations with a lower percent holding positive perceptions of health, a higher percent experiencing delays in receiving health care, and a lower percent with full-time employment.

These findings contrast with those of a recent report where Asian Pacific Islander (API) women residing in ethnic enclaves had better pregnancy outcomes than API women residing in non-enclave areas (46). Culturally appropriate resources and reduced exposure to discrimination may promote health for pregnant API women living in ethnic enclaves (46). Similarly, a study of older Chinese Americans in Chicago found evidence for a protective effect on oral health from living in areas with high neighborhood cohesion, particularly in ethnic enclaves such as Chinatown (47). Another study found that nativity and length of time living in the U.S. could be a mediating factor in determining whether ethnic enclaves alleviate or amplify experiences of stress and discrimination for Asian American women (48).

There were several limitations in this study. First, the data from community health surveys were only available at the community district level. Although we downscaled the health data from the community district level to the Census tract level (a smaller spatial unit), this process could lead to misaligned data and information loss. Second, counter to expectations, the study found only weak evidence of associations between living in a Chinese American ethnic enclave and health-related behaviors such as smoking and consumption of sugary beverages. This finding might be attributable to the limited number of demographic control variables and the measurement error from the health behaviors being measured at a different level of aggregation than the demographic and population data. Since the influence of ethnic enclaves on health is complex, understanding nuanced distinctions between demographic groups is critical to ascertaining whether ethnic enclaves are associated with positive or negative health outcomes for a given community context.

## Conclusion

This study examined changes in residential settlement patterns for Chinese Americans living in New York City using spatial and temporal analyses. This process involved the development of a two-dimensional method, using both sociodemographic and geographic characteristics, to identify ethnic enclaves. Correlation analysis indicated that living in a Chinese American ethnic enclave was associated with more negative self-perception of health, longer delays in receiving health care, less full-time employment, higher poverty rate, and less educational attainment. Results of the time series analysis indicated that Chinese Americans were strongly clustered in New York City in a manner where they were segregated with other Asian Americans away from other minority (non-White) ethnic groups, even though the extent of this segregation decreased from 2000-2016. Fundamentally, the findings of this study reveal that Chinese Americans living in ethnic enclaves face substantial barriers to achieving health equity when compared with the majority (White) population.

## Data Availability Statement

The original contributions presented in the study are included in the article/Supplementary Material, further inquiries can be directed to the corresponding author.

## Author Contributions

QZ and SM contributed to conception and design of the study. HP and MN provided support for theory construction and statistical analysis. QZ wrote the first draft of the manuscript. All authors contributed to manuscript revision, read, and approved the submitted version.

## Funding

This research was conducted as part of a study titled, Implementing a Participatory, Multi-level Intervention to Improve Asian American Health, that was supported by the National Institute of Dental and Craniofacial Research of the U.S. National Institutes of Health (Grant No. U56-DE027447) to MN, SM, and Trinh-Shevrin (MPIs). Publishing was also supported by the Natural Science Foundation of Fujian Province (Grant No. 2021J05220) titled Research on the Spatial Features and Guidance and Control Strategies of Urban Human Settlement Environment Health and Efficiency.

## Conflict of Interest

The authors declare that the research was conducted in the absence of any commercial or financial relationships that could be construed as a potential conflict of interest.

## Publisher's Note

All claims expressed in this article are solely those of the authors and do not necessarily represent those of their affiliated organizations, or those of the publisher, the editors and the reviewers. Any product that may be evaluated in this article, or claim that may be made by its manufacturer, is not guaranteed or endorsed by the publisher.

## Appendix

**Table:** The table below provides a full description of the variables used in this study.

**Variable**	**Description**	**Source**
**Main independent variables**		
% With limited english	Percent of speak English less than “very well”	2016 ACS
Age groups	Population by age cohort: Under 5, 5–9, 10–14, 15–24, 25–34, 35–44, 45–54, 55–64, 65–74, 75–84, 85 years and over.	2016 ACS
Median age	Median age (years)	2016 ACS
% In poverty	Percent of total population living in poverty	2016 ACS
% With full time employment	Percent of total population that usually worked 35 or more hours per week	2016 ACS
% Foreign born	Percent of total population estimated to be foreign born	Modified from 2016 ACS
% 25–64 years	Percent of total population aged 25 to 64 years	Modified from 2016 ACS
% 65+ years	Percent of total population aged 65 years and over	Modified from 2016 ACS
% 25–64 years with < HS education	Percent of population aged 25 to 64 years with less than a high school education	Modified from 2016 ACS
% 25–64 years with < HS education & private ins.	Percent of population aged 25 to 64 years with less than a high school education who have private health insurance	Modified from 2016 ACS
% 25–64 years with < HS education & public ins.	Percent of population aged 25 to 64 years with less than a high school education who have public insurance coverage	Modified from 2016 ACS
% 25–64 years with < HS education & no ins.	Percent of population aged 25 to 64 years with less than a high school education who have no health insurance coverage	Modified from 2016 ACS
% 65+ years with < HS education	Percent of population 65 years and over with less than a high school education	Modified from 2016 ACS
% 65+ years with < HS education & private ins.	Percent of population 65 years and over with less than a high school education who have private health insurance	Modified from 2016 ACS
% 65+ years with < HS education & public ins.	Percent of population 65 years and over with less than a high school education who have public insurance coverage	Modified from 2016 ACS
% 65+ years with < HS education & no ins.	Percent of population 65 years and over with less than a high school education who have health insurance coverage	Modified from 2016 ACS
**Community Health Indicators**		
% With good perception of health	Age-adjusted percent of adults reporting that their health is “excellent,” “very good,” or “good” on a 5-level scale (Poor, Fair, Good, Very Good or Excellent)	2011-2013 NYC DOHMH, Community Health Survey
% Who smoke tobacco	Age-adjusted percent of adults that reported being a current tobacco smoker	2011-2013 NYC DOHMH, Community Health Survey
% Who consume sugary drinks	Age-adjusted percent of adults that reported drinking one or more 12 ounce sugar-sweetened beverages (sodas, iced tea, sports drinks, etc.) per day	2011-2013 NYC DOHMH, Community Health Survey
% With delays in receiving health care	Age-adjusted percent of adults that reported needing medical care in the past 12 months but did not receive it	2011-2013 NYC DOHMH, Community Health Survey
**Controls**		
Population	Population in Census tract *i* in year 2016	2016 ACS
% Chinese Americans	Percent of Chinese Americans in Census tract *i* in year 2016	2016 ACS
% White	Percent of white population in Census tract *i* in year 2016	2016 ACS

*Notation: 2016 ACS: U.S. Census Bureau, 2012-2016. American Community Survey 5-Year Estimates. 2011-2013 NYC DOHMH: New York City Department of Health and Mental Hygiene, Community Health Survey, 2011-2013*.
